# Testing the Cow’s Milk-Related Symptom Score (CoMiSS^TM^) for the Response to a Cow’s Milk-Free Diet in Infants: A Prospective Study

**DOI:** 10.3390/nu11102402

**Published:** 2019-10-08

**Authors:** Silvia Salvatore, Elisabetta Bertoni, Federica Bogni, Valentina Bonaita, Chiara Armano, Alex Moretti, Mario Baù, Chiara Luini, Enza D’Auria, Maddalena Marinoni, GianVincenzo Zuccotti, Massimo Agosti

**Affiliations:** 1 Department of Pediatrics, Ospedale F. Del Ponte, ASST-Sette Laghi, Università dell’Insubria, 21100 Varese, Italy; silvia.salvatore@uninsubria.it (S.S.); Bertonielisabetta93@gmail.com (E.B.); valebonaita@gmail.com (V.B.); chiara.armano86@gmail.com (C.A.); alexmoretti.med@gmail.com (A.M.); mario.bau@live.it (M.B.); chiara.luini@asst-settelaghi.it (C.L.); maddalena.marinoni@asst-settelaghi.it (M.M.); 2 Statistic and Economic Science, University of Bicocca, 20126 Milano, Italy; Federica.bogni@gmail.com; 3 Department of Pediatrics, Vittore Buzzi Children’s Hospital-University of Milan, 20154 Milan, Italy; enza.dauria@unimi.it (E.D.);

**Keywords:** cow’s milk allergy, regurgitation, CoMiSS^TM^, cow’s milk-free diet, infants, crying, hydrolysed formulas, gastrointestinal

## Abstract

The diagnosis of cow’s milk allergy (CMA) is particularly challenging in infants, especially with non-Immunoglobulin E (IgE)-mediated manifestations, and inaccurate diagnosis may lead to unnecessary dietary restrictions. The aim of this study was to assess the accuracy of the cow’s milk-related symptom score (CoMiSS^TM^) in response to a cow’s milk-free diet (CMFD). We prospectively recruited 47 infants (median age three months) who had been placed on a CMFD due to persisting unexplained gastrointestinal symptoms. We compared data with 94 healthy controls (median age three months). The CoMiSS^TM^ score was completed at recruitment and while on the exclusion diet. In 19/47 (40%) cases a response to the diet occurred. At recruitment CoMiSS^TM^ was significantly higher in cases compared to controls (median score 8 vs. 3; *p*-value: <0.05), 9 cases had a score ≥12 and 8/9 normalized on CMFD. An oral milk challenge was performed in all 19 responders and six of these had a positive reaction to cow’s milk (CM). In eight infants IgE allergy tests were positive. The receiver operation characteristic (ROC) curve identified a CoMISS^TM^ score of 9 to be the best cut-off value (84% sensitivity, 85% specificity, 80% positive (PPV) and 88% negative predictive value (NPV)) for the response to CMFD. We found CoMiSS^TM^ to be a useful tool to help identify infants with persisting gastrointestinal symptoms and suspected CMA that would benefit from CMFD.

## 1. Introduction

Cow’s milk (CM) protein allergy (CMA) is an immune reaction to specific CM proteins occurring in 2–5% of infants, presenting with skin, gastrointestinal (GI) and/or respiratory symptoms [1,2]. These manifestations can be acute, and in rare cases even life threatening, such as anaphylaxis, or can be chronic. In infants with gastrointestinal symptoms, the diagnosis of CMA is particularly challenging due to the lack of optimal diagnostic tests, lack of biomarkers and a broad spectrum of presenting symptoms [3,4,5]. Several risk factors have been identified for CMA, but its pathogenesis and clinical correlation still needs to be fully clarified for both breast- and formula-fed infants [6,7,8,9]. A cow’s milk-free diet (CMFD) is a recognized treatment option for infants with diagnosed CMA [1,2,5,10]. However, not all GI symptoms are allergic in nature. Regurgitation, excessive crying or colic, for example, are common physiological conditions, often showing spontaneous resolution in the first months of life. An oral food challenge (OFC) is considered necessary to confirm the diagnosis of CMA as well as the acquisition of tolerance [1,2,5,10]. However, it is often difficult to interpret, particularly in children with non-IgE-mediated CMA, which may take days or weeks to diagnose, or may be refused by parents who fear severe reactions [5,10,11].

For these reasons, different nutritional and pharmacological tests have been employed to try to improve the diagnosis [4,10,12,13]. The real challenge for the clinician is how to rapidly identify subjects who may benefit from CMFD when CMA is suspected. In 2015 a Belgian group created a symptom-based score system [14] to help in the identification of infants with CMA, especially those with non-IgE mediated forms. Crying, regurgitation, stool pattern, skin and respiratory symptoms were all considered, and an arbitrary cut-off of 12 points was suggested as a possible score to indicate CMA [14]. The authors introduced the acronym “CoMiSS^TM^” (cow’s milk-related symptom score awareness tool) [15]. The score was assessed in symptomatic infants (aged two weeks to six months) at initial diagnosis of CMA, and later when placed on the CMFD [15]. OFC was positive in 80% of infants in which CoMiSS^TM^ decreased to ≤6 after one month of elimination diet [15]. Since then, four other reports confirmed the high predictive value of CoMiSS^TM^ in relation to the CM OFC; the reduction of the score to <6 was also associated with the response to CMFD [16,17,18,19]. Very recently, an international study tested the score in a population of healthy infants and found a median value of 3 [20]. 

We aimed to assess the accuracy of CoMiSS^TM^ in identifying infants with gastrointestinal symptoms who benefit from CMFD.

## 2. Methods

### 2.1. Design of the Study

This was an open prospective study assessing CoMiSS^TM^ in infants (aged between 1 and 12 months) who started CMFD due to acute or chronic symptoms or gastrointestinal symptoms suspected for CMA. 

CoMiSS^TM^ is a simple tool that rates five different symptoms: Daily duration of crying, number and volume of episodes of regurgitation, consistency of stools, presence and severity of atopic eczema or urticaria and presence and severity of respiratory symptoms (supplementary File S1). CoMiSS^TM^ ranges from 0 to 33 points: Crying, regurgitation and cutaneous symptoms are scored from 0 to 6, with each increase of 1 point meaning more severe symptoms, up to 6 points as the worst symptom; respiratory symptoms are scored from 0 to 3, with 0 as no symptom, 1 mild, 2 moderate and 3 severe. Stool consistency is scored based on the Bristol stool scale with 0 for normal stools (type 3 and 4), 2 points for soft stools (type 5), 4 points for hard stools (type 1 and 2) or liquid stools (type 6) and 6 points for watery stools (type 7). 

The primary outcome is to establish the sensitivity and specificity of CoMiSS^TM^ in identifying infants who respond to CMFD. The secondary outcomes include the evaluation of the score in healthy infants and to identify the best cut-off score in the symptomatic population. 

This was an independent study in all stages of the design and conduction, collection, management, analysis or interpretation of the data, preparation, review and approval of the paper. We downloaded the CoMiSS^TM^ tool from the dedicated website.

All parents of enrolled infants signed a written consent and the study was approved by our Institutional Review Board. 

### 2.2. Study Population

From October 2017 to June 2018 we recruited infants referred to our pediatric gastroenterology clinic who had started a CMFD due to persistent unexplained gastrointestinal symptoms (such as vomiting, regurgitation, constipation or diarrhea) and/or other manifestations (such as failure to thrive, crying/fussiness, sleeping problems, dermatitis and feeding refusal). Infants presenting with unequivocal and/or severe acute symptoms after introduction of CM protein who also had positive IgE test results were prescribed a CMFD. In all other infants, parental reassurance, behavior management and nutritional advice (adequate intake and thickening formulas in regurgitating infants) were first attempted for at least one week. CMFD was started if a clear improvement, as perceived by parents, did not occur. Infants were excluded if they were older than 12 months of age, already on CMFD, on enteral tube feeding, neurologically impaired, had gastrointestinal malformations or surgery or were on anti-reflux medication. 

### 2.3. CoMiSS^TM^ Evaluation

Based on the infant’s clinical history, CMFD was introduced by an expert pediatric gastroenterologist (SS) blind to the CoMiSS^TM^. The score was completed by another clinician helping parents of recruited infants at the first clinical evaluation (T0) after 2–4 weeks of diet (T1) and, eventually, after oral challenge (T2). Age, gender, dietary intake, results of allergy tests and family history of allergy were also recorded.

We defined a positive score when CoMiSS^TM^ was ≥12 (as originally proposed [14]) and a negative score when it was <12. Based on parents’ reports, we calculated any variation of the score on diet and the number of infants who switched from a positive to a negative CoMiSS^TM^. We also compared the CoMiSS^TM^ score of symptomatic infants with the score obtained (only at enrollment, T0) in a control group of infants referred for a minor trauma, not on diet or on anti-reflux medication and perceived by parents as healthy infants. To limit the possible effect of physiologic improvement of symptoms with time we created a mathematical model to rate the response to CMFD that was defined as having a score that decreased by least 50% from T0 and below the median value of the control population. To limit a possible false negative result from the CoMiSS^TM^ we analyzed also infants who had had a negative score at first visit. 

### 2.4. CMFD

In formula-fed infants, hypoallergenic formulae were used for the management of CMA, while in breast-fed infants, maternal CMFD was recommended and continuation of breast-feeding. In this study infants on exclusive formula-feeding were prescribed either an amino acid formula (AAF), an extensively hydrolysed formula (eHF) or a rice-based hydrolysate formula (RHF). In infants already weaned, CM protein was excluded from their complementary feeds.

### 2.5. Allergy Tests and OFC

In all infants who commenced on the CMFD, allergy tests were performed and an open food challenge took place within three months following inclusion. Skin prick tests were performed on the infant’s volar forearm using a 1-mm disposable lancet, a standard CM-based formula (prick to prick test) as well as histamine, a saline solution (negative control) and CM-protein allergens manufactured by ALK, Copenhagen, Denmark. After 15 min the maximal diameter of the wheal and flare were recorded. A positive skin prick test result was considered when the diameter of the wheal was larger than 3 mm compared to the negative (saline) control.

The open CM challenge was performed in our hospital according to the previous consensus report [21] and guidelines [5,10] and was interpreted by two experienced clinicians (MM and CL) who were blinded to the study and to the CoMiSS^TM^ score. For the OFC, the protocol was based on semi-logarithmic incremental doses of CM protein (3, 10, 30, 100, 300, 1000 and 3000 mg) at intervals of 20 min [21]. The infant was observed for an additional 2 h in the hospital after the last dose was administered, while being monitored for any reaction. Acute reactions were defined as those occurring within 2 h of the last dose of CM during the challenge. In the absence of an acute reaction, the parents were instructed to give the infant at least 250 mL per day of a standard CM-protein based formula at home, starting the following morning for 14 days [16]. During this period parents continued to monitor symptoms, informing the clinician in cases of occurrence of symptoms including gastrointestinal, cutaneous, respiratory or general. Delayed reactions were considered up to 2 weeks from the OFC in the absence of other possible confounding effects (i.e., infection). A positive OFC was considered when at least one of the following symptoms occurred: Urticaria (>3 hives), severe lip or face edema, generalized erythema, persistent sneezing or rhinorrhea or dry cough, hoarseness, wheezing or stridor, at least two episodes of vomiting or loose stools, altered mental status /hyporeactivity or cardiovascular collapse [21].

### 2.6. Statistical Analysis

Demographic data, symptoms, CoMiSS^TM^ scores, results of the allergy tests and of the OFC, the type of CMFD and family history of allergy were all recorded in an Excel database. An independent statistician performed the statistical analysis by R program (RStudio Version 1.0.136—© 2009–2016, RStudio, Inc., Boston, MA, US). Median values were considered whenever a non-normal distribution was present.

We compared the CoMiSS^TM^ distribution and the median score at enrollment in cases and controls (T0) and when off and on CMFD in cases (T1). 

For statistical evaluation, responsive to CMFD’ was defined as all infants who passed from a CoMiSS^TM^ score above the median of the symptomatic population at T0 to a score below the median of the control population at T1. We also studied the normalization of the score, i.e., the transition of CoMiSS^TM^ from T0 ≥12 to T1 <12. 

We used the Wilcoxon test as non-parametric statistical test and we analyzed the CoMiSS^TM^ score at T0 and T1; significance was set at *p* value <0.05. We calculated the sensitivity, specificity and positive (PPV) and negative predictive value (NPV) and created a ROC-curve to identify the best cut-off for CoMiSS^TM^ to predict the response to CMFD.

The sample size calculation was based on a previous study where CoMiSS^TM^ was used to assess symptoms in infants with CMA who had been prescribed an extensively hydrolyzed formula. In this study 29 infants were included [16]. As healthy infants were evaluated just once and symptomatic infants at least twice, we arbitrarily fixed that the number of the control population should double the number of cases. 

## 3. Results

We recruited 47 infants who had commenced on a CMFD (26 male, median age 3 month, range 10 days–8 months) and 94 healthy controls (43 male, median age 3 months, range 15 days–8 months). At recruitment (T0) the median score of CoMiSS^TM^ was 8 (range 2–16) in cases and 3 (range 0–11) in controls. 

### 3.1. CoMiSS^TM^ Evaluation 

In the control group the vast majority of the population had a score <6, 34% between 0 and 1 and 45% of controls between 2 and 6. The score was significantly lower than in the CMA group (*p* < 0.05).

**Figure 1:**
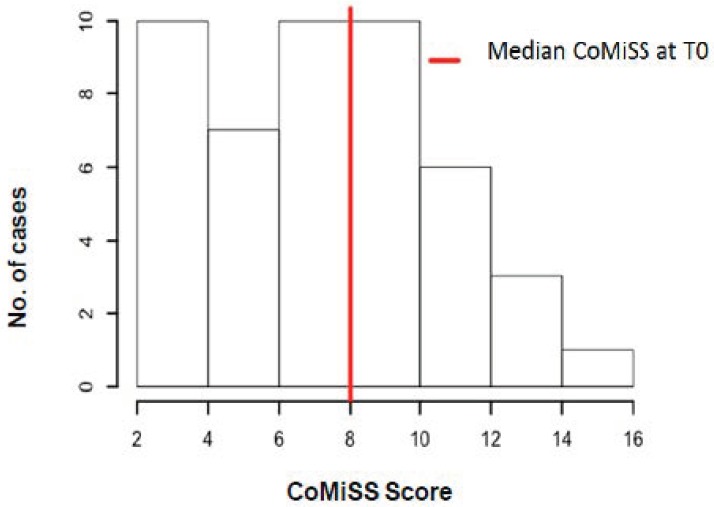
Distribution of the cow’s milk-related symptom score (CoMiSS^TM^) in symptomatic infants. T0: Time 0 (first visit).

**Figure 2:**
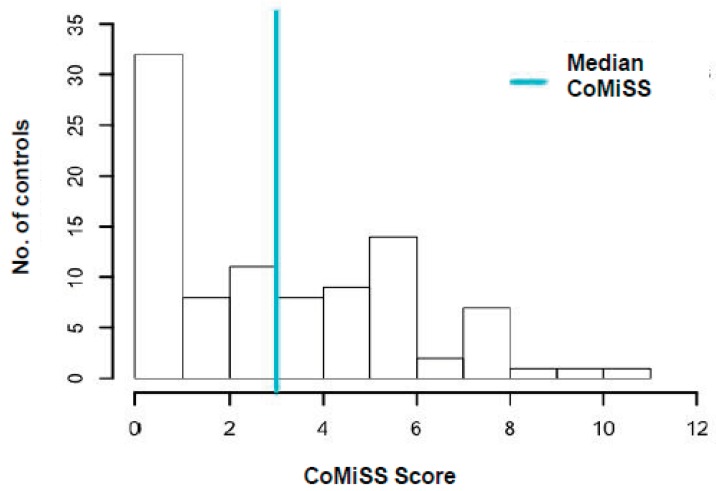
Distribution of the CoMiSS^TM^ score in controls. T0: Time 0 (first visit).

The distribution of the CoMiSS^TM^ score at T0 in cases and controls is shown in Figure 1 and Figure 2. 

The symptoms included in CoMiSS^TM^ and differences between cases and controls at T0 are reported in Table 1. 

### 3.2. Clinical Presentation

In our population the most frequent symptoms were crying and regurgitation both reported in 75% of cases. Unexplained rectal bleeding was reported in four infants (8%) and was associated with other symptoms.

Seven (15%) infants had symptoms such as vomiting and/or urticaria that occurred acutely after CM protein exposure, but none had anaphylaxis or required adrenalin. CoMiSS^TM^ was not significantly different (*p* = 0.31) in infants with acute or chronic symptoms (median 8 vs. 7.5, range 6–13 vs. 2–15). 

**Figure 3:**
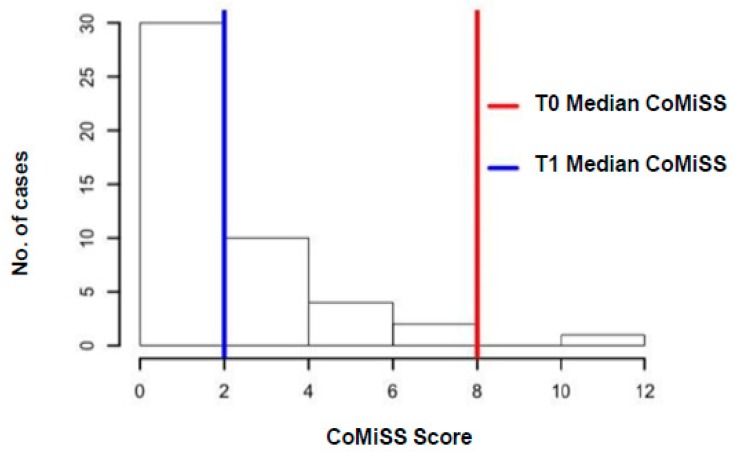
Decrease of the CoMiSS^TM^ score in cases on cow‘s milk-free diet (CMFD) (T1). T0: Time 0 (first visit); T1: Time 1 (second visit after 2–4 weeks of cow‘s milk (CM) protein elimination diet).

Parents reported a reduction of symptoms in infants on the CMFD in 39 (83%) of cases; 19 (40%) had a significant response to CMFD as defined above. The decrease of the CoMiSS^TM^ score in cases after 2–4 weeks of cow‘s milk-free diet (CMFD) is shown in Figure 3. 

Differences between responders and non-responders to CMFD are reported in Table 2.

**Figure 4:**
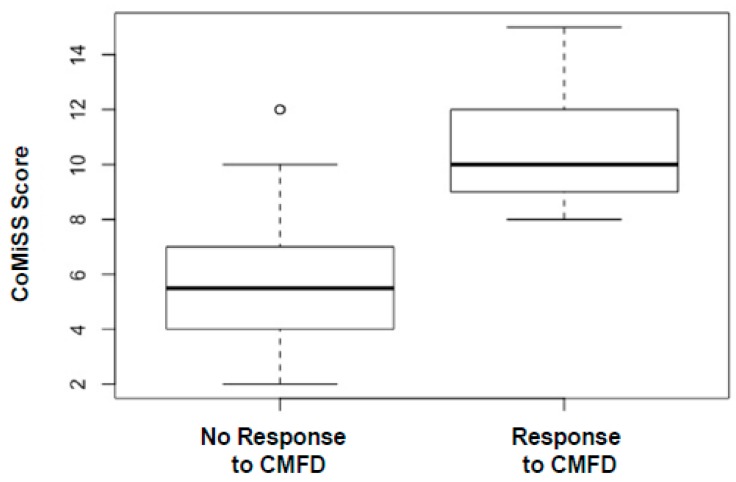
Box plot distribution of CoMiSS^TM^ at baseline comparing infants who responded and did not respond to CMFD.

The median score of CoMiSS^TM^ was significantly higher in infants who responded to the diet compared to the ones who did not respond (see Figure 4).

Only nine of the 47 infants (19%) who started CMFD had a CoMiSS^TM^ score ≥12 at T0, and eight out of nine (89%) had a negative (<12) score at T1. One infant who did not achieve a negative score at T1 was subsequently diagnosed with gastroesophageal reflux disease, based on a pathological impedance-pH monitoring, which improved on acid inhibitors.

### 3.3. Diet

The majority of infants on CMFD were prescribed an extensively hydrolyzed CM protein formula (29/47, 62%). 11% (five infants) were taking an amino acid-based formula due to more severe or acute reactions and 10% (two infants) of infants were taking a rice-based hydrolyzed formula. A maternal diet was started in seven infants, exclusively breast-fed (7/47, 15%), and was associated to CM protein exclusion in four breast-fed infants already having complementary feeding. At T1 the median CoMiSS^TM^ decreased more in infants on amino acid-based formulas compared to the ones on extensively hydrolyzed formulas (−8.5 points vs. −4.5 points).

Eight infants (17%) showed positive allergy tests to CM proteins. Among the 39 infants whose parents reported an improvement of symptoms on CMFD, OFC was performed in 21 infants (54%), and in all the 19 infants who showed a statistical response to CMFD, as defined above. The OFC was carried our after 2–10 (mean 6 ± 4.2) months from starting the elimination diet: Three patients had acute reactions and three reported the recurrence of symptoms in the following days. The other 15/21 (71%) infants had negative OFC. The median age at OFC was 9 months (SD ± 4 months). 

### 3.4. Family History

Family history of allergy was reported in 24/47 (51%) infants and the median CoMiSS^TM^ in this group did not differ from the one obtained in the 23 infants with a negative family history (8.5 vs. 7, *p* = 0.35). However, family history of allergy significantly influenced the response to CMFD; 14 of the 19 responsive infants (74%) had a family history of allergy compared to 10 of the 28 infants (36%) who did not respond (*p* = 0.02).

### 3.5. CoMiSS^TM^ Cut-off

Only 7/19 of the infants who responded to CMFD (37%) had a T0 CoMiSS^TM^ score ≥12, and the other 12 infants (63%) had a T0 CoMiSS^TM^ score <12. Only two of the infants who did not respond to the diet had a T0 CoMiSS^TM^ score ≥12. Applying the cut-off score of 12 we obtained a sensitivity of 0.37 (7/19) and a specificity of 0.92 (26/28); the PPV was 0.77 (7/9) and the NPV was 0.68 (26/38). 

**Figure 5:**
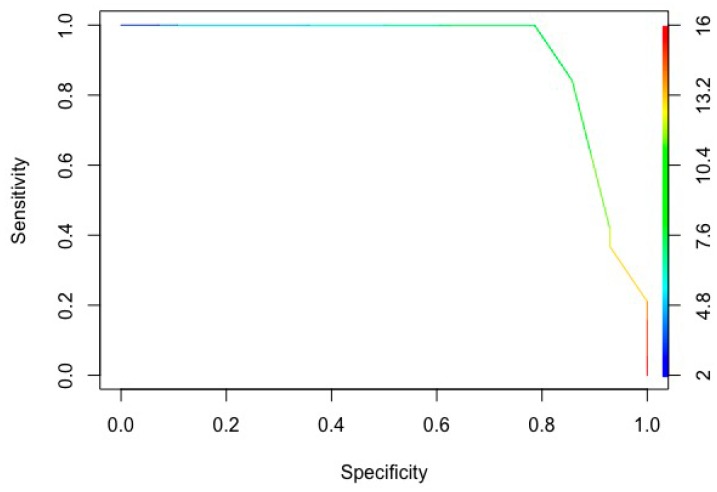
ROC curve of the CoMiSS^TM^ score.

The ROC curve identified the score of 9 as the best cut-off for the test (area under the curve 0.91): 91% of the real positive infants (responsive to CMFD infants with a positive test) showed a significantly higher score than the real negative non-responsive infants (see Figure 5). 

The sensitivity of the test with a cut-off of 9 was 0.84 (16/19), and the specificity 0.85 (24/28), the positive predictive value (PPV) was 0.8 (16/20) and the negative predictive value (NPV) was 0.88 (24/27). 

## 4. Discussion

In our population of infants referred to the GI clinic with persistent GI symptoms suspected to be related to CMA, a positive CoMiSS^TM^ score appeared to predict those infants who later responded to CMFD. A score of 9 could better predict those who would respond to the CMFD instead of 12, as previously suggested. The CoMiSS^TM^ score was significantly different between cases and controls and between those off or on CMFD. Most infants who benefited from CMFD showed more than one symptom, with crying and regurgitation being the most frequently reported but neither necessary nor sufficient to predict the response to the elimination diet. The group of infants who responded to the CMFD with a reduction of CoMiSS^TM^ below the median of the control population included both infants with IgE positive and negative test results and also breast-fed infants. 

The diagnosis of CMA in infants is often challenging, particularly in cases with chronic gastrointestinal symptoms and negative allergy tests [1,2,3,4,5,10]. No symptom or sign alone is specific for the diagnosis of CMA or predictive of a response to CMFD [4,5,10,11]. Clinical presentation may overlap with functional gastrointestinal disorders, gastroesophageal reflux disease, infections or other conditions [4,12,22,23]. OFC is considered the gold standard method to diagnose CMA [1,2,5,10], but it is often refused by parents of young infants or delayed due to fear of a severe reaction or for other reasons. Hence, when OFC is performed the infants could have naturally resolved their symptoms through and acquired CM tolerance. Furthermore, the clinical reaction during or after an OFC is sometimes difficult to interpret, especially in infants, and is possibly related to mechanisms other than immune activation [4,11,22,23,24]. Protein hydrolysis accelerates gastric emptying time, improving motility or gastroesophageal-related symptoms [4,11,12,23]. In addition, the absence or reduction of lactose content may reduce intestinal gas, the fermentation process, crying and diarrhea in a subgroup of patients [4,12]. As a result, in infants with gastrointestinal symptoms, CMA may be over-diagnosed and CMFD unnecessarily prolonged [23,24]. Nevertheless, acid inhibitors are often inappropriately started for symptoms that could improve on CMFD [12]. For these reasons, it is necessary to find a handy clinical tool that identifies infants who can benefit from a CMFD, and that can monitor the efficacy of the elimination diet [24]. 

CoMiSS^TM^ has recently been introduced as an “awareness tool” for CM-related symptoms [14,15]. In the last years different authors reported a good correlation between a positive (score ≥12) CoMiSS^TM^ and a positive OFC and showed a reduction of the score on CMFD [16,17,18,19]. Very recently, an international study tested the specificity of CoMiSS^TM^ in a population of healthy infants and found a median value of 3 [20]. 

We found the same median score using CoMiSS^TM^ in our healthy control group, with none of these healthy infants scoring above 11. Different from previous studies, we also included symptomatic infants with a negative CoMiSS^TM^. In our population, the ROC curve identified the cut-off of 9 as the most accurate score, with a significant increased sensitivity and negative predictive value, a similar positive predictive value and a just slightly reduced specificity of the test compared to the cut-off of 12 (from 92% to 85%). 

The main strength of our study was that we compared the CoMiSS^TM^ score in a group of symptomatic infants with a group of healthy age matched controls. In addition, we could analyze the accuracy of the score in a group of infants with prevalent non-IgE manifestations before and during CMFD and, in the ones who benefited from the diet, also after the challenge. We also collected additional symptoms possibly related to CMA such as rectal bleeding [22] and feeding and sleeping problems. However, we did not find any significant correlation, possibly due to the limited number of patients. 

Family history of allergy did not influence the clinical presentation in our cohort but was significantly more common in infants who responded to CMFD. The major limitation of this study was due to its open design, with the CoMiSS^TM^ scored based on parental reporting and with the performance of an open OFC only in some patients. To reduce these potential flaws both the clinician who prescribed the CMFD and the clinician who interpreted the OFC were unaware (blind) of the CoMiSS^TM^ score. However, we could not exclude a placebo effect of CMFD or a physiologic improvement of symptoms with the progression of time. To limit these effects we compared the CoMiSS^TM^ score in symptomatic infants with the CoMiSS^TM^ score of healthy infants. In addition we constructed a mathematical model to rate a (significant) response to CMFD at a score value of 2, lower than the median score of our control population. This score was largely below the positive CoMiSS^TM^ score of 12 and the cut-off reduction of <6, as originally proposed. Forty per cent of our population significantly responded to CMFD and different types of elimination diet were well tolerated and continued despite the palatability, considered poor, of the extensive hydrolyzed and the amino acid-based formulas [25]. However, OFC was performed only in half of the enrolled population and, in most cases, delayed beyond the scheduled time, which may have impacted our results. OFC was positive in 29% of infants who responded to CMFD, which might suggest that tolerance was already acquired, or the CMFC was unnecessary and prolonged. As we only followed up on the symptomatic infants on CMFD we could analyze neither the possible placebo effect of the elimination diet nor the natural evolution of the reported symptoms. However, for ethical, medical and economic reasons, a CMFD and a subsequent OFC could not be proposed to healthy infants without marked or persistent gastrointestinal symptoms. Our group of patients was also heterogeneous in terms of CMFD with 11/47 (23%) being breast-fed. The small sample size did not allow us to analyze the different types of diet or to generalize our results for infants with different clinical presentations. Moreover, we did not consider ethnicity, psychosocial and economical factors all of which may influence the parental report of symptoms and CoMiSS^TM^ results. 

To date, in infants with chronic symptoms and negative allergy tests, CMFD is often started based on a subjective interpretation of clinical history and clinicians‘ attitudes and experience [24]. If our results will be be confirmed in a larger population, CoMiSS^TM^ could be introduced into clinical practice as a valuable tool for thedetection of infants with GI symptoms who should start CMFD and could be used to monitor these infants during their period of CM exclusion, and possibly during the reintroduction of CM protein. 

The improvement of symptoms with targeted dietary treatment could also reduce the stress level of caregivers and the healthcare costs and improve the quality of life of food-allergic infants and family [12,13,24].

## 5. Conclusions

CoMiSS^TM^ can be a helpful tool to identify which infants with persisting gastrointestinal symptoms would benefit from CMFD when CMA is suspected. Our findings support the use of CoMiSS^TM^ in both IgE positive and negative infants; we propose a lower cut-off score of 9 to improve the accuracy of the test. However, our results need validation by other groups before routine use of CoMiSS^TM^ is recommended in clinical practice.

## Figures and Tables

**Table 1:** Comparison of CoMiSS^TM^ items and scores between cases and controls.

	Crying			Regurgitation			Stools (Bristol Scale) *			Skin Symptoms			Respiratory Symptoms		
Group	Cases *N* (%)	Controls *N* (%)	*p*	Cases *N* (%)	Controls *N* (%)	*P*	Cases *N* (%)	Controls *N* (%)	*P*	Cases *N* (%)	Controls *N* (%)	*P*	Cases *N* (%)	Controls *N* (%)	*P*
YES	35 (75)	52 (55)	N.S.	35 (75)	57 (61)	N.S.	20 (43)	35 (37)	N.S.	18 (38)	14 (15)	0.04	20 (43)	18 (19)	N.S.
NO	12 (25)	42 (45)		12 (25)	37 (39)		27 (57)	59 63)		29 (62)	80 (85)		27 (57)	76 (81)	
TOTAL	47(100)	94 (100)	-	47(100)	94(100)	-	47(100)	94(100)	-	47(100)	94(100)	-	47(100)	94(100)	-
Score															
0	12 (26)	42 (45)	N.S.	12 (26)	37 (39)	N.S.	27 (57)	59 (63)	N.S.	29 (62)	80 (85)	0.04	27 (57)	76 (81)	N.S.
1	5 (11)	25 (27)	N.S.	8 (17)	30 (32)	N.S.	n.a.	n.a.	n.a.	5 (11)	6 (6)	N.S.	12 (26)	13 (14)	N.S.
2	9 (19)	17 (18)	N.S.	11 (23)	17 (18)	N.S.	5 (11)	28 (30)	N.S.	4 (8)	6 (6)	N.S.	8 (17)	4 (4)	N.S.
3	8 (17)	9 (10)	N.S.	4 (8)	10 (11)	N.S.	n.a.	n.a.	n.a.	1 (2)	0	N.S.	0	1 (1)	N.S.
4	7 (15)	1 (1)	0.02	5 (11)	0	0.03	15 (32)	7 (7)	0.01	2 (4)	2 (2)	N.S.	n.a.	n.a.	n.a.
5	3 (6)	0	N.S.	2 (4)	0	N.S.	n.a.	n.a.	n.a.	0	0	-	n.a.	n.a.	n.a.
6	3 (6)	0	N.S.	5 (11)	0	0.03	0	0	-	6 (13)	0	0.01	n.a.	n.a.	n.a.

Legend: *N* = number of infants; % = percentage; YES was considered when the symptom was reported; * for Stools YES was considered when Bristol Scale was ≠ 0; N.S. was considered when *p* > 0.05; n.a.: Not applicable because the CoMiSS^TM^ does not include this point score for this item in the Bristol scale or in the respiratory symptoms.

**Table 2:** Comparison between infants with response or no response to CMFD.

	RESPONSE to CMFD		NO RESPONSE to CMFD		*p*-Value
	*N*	(%)	*N*	(%)	
Number of cases	19	(40)	28	(60)	N.S.
Median age at enrollment	3 mo		3 mo		N.S.
Family history of allergy	14	(74)	10	(36)	0.02
Positive SPT	5	(26)	3	(11)	N.S.
Breast Milk (BM) Exclusive BM BM + formula BM +complementary feeding	5 3 0 2	(26) (16) (0) (10)	6 4 0 2	(21) (14) (0) (7)	N.S. N.S. - N.S.
Formula Fed - AAF - eHF - RiceHF	14 2 10 2	(74) (10) (53) (10)	22 3 19 0	(79) (11) (68) (0)	N.S. N.S. N.S. N.S.
Median CoMiSS at T0	10		5.5		0.01
CoMiSS ≥12 at T0	7	(37)	2	(7)	0.03
Symptoms	median score	mean score ± SD	median score	mean score ± SD	
cry	2	2.7 ± 1.9	2	2.1 ±1.9	N.S.
regurgitation	2	2.3 ± 1.9	2	1.8 ± 1.9	N.S.
stools	0	2.6 ± 1.9	0	0.78 ± 1.8	N.S.
skin symptoms	0	2.4 ± 2.2	0	0.6 ±2.2	N.S.
respiratory symptoms	0	0.6 ± 0.7	0	0.6 ± 0.78	N.S.
Acute symptoms	4	(21)	3	(11)	N.S.
Chronic symptoms	15	(79)	25	(89)	N.S.

## Supplementary Materials

The following is available online at https://www.mdpi.com/2072-6643/11/10/2402/s1, File S1: CoMiSS^TM^: Cow’s Milk-related Symptom Score Form.
