# Supplementary material for: Testing the Cow’s Milk-Related Symptom Score (CoMiSSTM) for the Response to a Cow’s Milk-Free Diet in Infants: A Prospective Study
Source: Nutrients. 2019 Oct 8;11(10):2402. doi: 10.3390/nu11102402 (PMC6835327; doi:10.3390/nu11102402)
Supplement: Supplementary file 1 [file nutrients-11-02402-s001.pdf]

CoMiSS: Cow's Milk-related Symptom Score

Last name:	First name:	Age:	Date:
....	....	....	....

PURPOSE

The CoMiSS is a simple, fast and easy-to-use awareness tool for cow's milk-related symptoms. It increases awareness of the most common symptoms of cow's milk protein allergy (CMPA) that in turn can aid an earlier diagnosis. CoMiSS can also be used to evaluate and quantify the evolution of symptoms during a therapeutic intervention.

INSTRUCTIONS

If there is a suspicion of cow's milk-related symptoms, rate the observed/reported symptoms by choosing the most appropriate score for each type of symptom. Once completed, add the scores together and put the total in the box at the bottom of the scoring form.

SYMPTOM	SCORE				
**Crying***	0	≤ 1 hour/day			
	1	1 to 1.5 hours/day			
	2	1.5 to 2 hours/day			
	3	2 to 3 hours/day			
	4	3 to 4 hours/day			SCORE
	5	4 to 5 hours/day			
	6	≥ 5 hours/day			
**Regurgitation**	0	0 to 2 episodes/day			
	1	≥ 3 to ≤5 of small volume			
	2	> 5 episodes of >1 coffee spoon			
	3	> 5 episodes of ± half of the feeds in <half of the feeds			
	4	Continuous regurgitations of small volumes >30 min after each feed			
	5	Regurgitation of half to complete volume of a feed in at least half of the feeds			SCORE
	6	Regurgitation of the complete feed after each feeding			
**Stools** (Bristol scale)	4	Type 1 and 2 (hard stools)			
	0	Type 3 and 4 (normal stools)			
	2	Type 5 (soft stool)			SCORE
	4	Type 6 (liquid stool, if unrelated to infection)			
	6	Type 7 (watery stools)			
**Skin** symptoms	0 to 6	Atopic eczema	**HEAD-NECK-TRUNK**	**ARMS-HANDS-LEGS-FEET**	
		Absent	0	0	
		Mild	1	1	SCORE
		Moderate	2	2	
		Severe	3	3	
	0 or 6	Urticaria	**NO** 0	**YES** 6	
**Respiratory** symptoms	0	No respiratory symptoms			
	1	Slight symptoms			SCORE
	2	Mild symptoms			
	3	Severe symptoms			

* Crying only considered if the child has been crying for 1 week or more, assessed by the parents, without any other obvious cause.

TOTAL SCORE

READING THE RESULT

The scoring ranges from 0 to 33. Each symptom has a maximal score of 6, except respiratory symptoms where the maximal score is 3.

If final score ≥ 12, the symptoms are likely cow's milk related. This could potentially be CMPA.

If final score <12, the symptoms are less likely related to cow's milk. Look for other causes.

CMPA diagnosis can only be confirmed by an elimination diet followed by an oral food challenge.

Vandenplas, Y., Dupont, C., Eigenmann, P., Host, A., Kuitunen, M., Ribes-Koninck, C., Shah, N., Shamir, R., Staiano, A., Szajewska, H. and Von Berg, A. (2015), A workshop report on the development of the Cow's Milk-related Symptom Score awareness tool for young children. Acta Paediatrica. doi: 10.1111/apa.12902

NestléHealthScience

© Nestlé Health Science 2015
